# Evolution and Functional Characterisation of Melanopsins in a Deep-Sea Chimaera (Elephant Shark, *Callorhinchus milii*)

**DOI:** 10.1371/journal.pone.0051276

**Published:** 2012-12-14

**Authors:** Wayne I. L. Davies, Boon-Hui Tay, Lei Zheng, Janine A. Danks, Sydney Brenner, Russell G. Foster, Shaun P. Collin, Mark W. Hankins, Byrappa Venkatesh, David M. Hunt

**Affiliations:** 1 School of Animal Biology, University of Western Australia Oceans Institute and Lions Eye Institute, University of Western Australia, Perth, Western Australia, Australia; 2 Nuffield Laboratory of Ophthalmology, Nuffield Department of Clinical Neurosciences, University of Oxford, John Radcliffe Hospital, Oxford, United Kingdom; 3 Comparative Genomics Laboratory, Institute of Molecular and Cell Biology, Agency for Science, Technology, and Research, Biopolis, Singapore; 4 Comparative Endocrinology and Biochemistry Laboratory, School of Medical Sciences, Health Innovations Research Institute, Royal Melbourne Institute of Technology University, Victoria, Australia; National Institutes of Health/NICHD, United States of America

## Abstract

Non-visual photoreception in mammals is primarily mediated by two splice variants that derive from a single melanopsin (*OPN4M*) gene, whose expression is restricted to a subset of retinal ganglion cells. Physiologically, this sensory system regulates the photoentrainment of many biological rhythms, such as sleep via the melatonin endocrine system and pupil constriction. By contrast, melanopsin exists as two distinct lineages in non-mammals, *opn4m* and *opn4x,* and is broadly expressed in a wide range of tissue types, including the eye, brain, pineal gland and skin. Despite these findings, the evolution and function of melanopsin in early vertebrates are largely unknown. We, therefore, investigated the complement of opn4 classes present in the genome of a model deep-sea cartilaginous species, the elephant shark (*Callorhinchus milii*), as a representative vertebrate that resides at the base of the gnathostome (jawed vertebrate) lineage. We reveal that three melanopsin genes, *opn4m1*, *opn4m2* and *opn4x*, are expressed in multiple tissues of the elephant shark. The two *opn4m* genes are likely to have arisen as a result of a lineage-specific duplication, whereas “long” and “short” splice variants are generated from a single *opn4x* gene. By using a heterologous expression system, we suggest that these genes encode functional photopigments that exhibit both “invertebrate-like” bistable and classical “vertebrate-like” monostable biochemical characteristics. We discuss the evolution and function of these melanopsin pigments within the context of the diverse photic and ecological environments inhabited by this chimaerid holocephalan, as well as the origin of non-visual sensory systems in early vertebrates.

## Introduction

Extant vertebrates are divided into the jawless (Agnatha), comprising the lampreys and hagfishes, and the jawed (Gnathostomata) lineages, which include all of the remaining vertebrate classes, such as the cartilaginous fishes, the ray-finned fishes, the lobe-finned fishes and the tetrapods. The cartilaginous fishes (Chondrichthyes) are numerous (over 800 species) and occupy diverse ecological niches. They are split into the holocephalans (chimaeras) and the elasmobranchs (skates, sharks and rays) and, as they reside at the base of the gnathostome lineage, occupy an important phylogenetic position for studies on the evolution of sensory systems. Chondrichthyans share a common ancestor that diverged from the main vertebrate lineage about 450 million years ago (mya) [1], with chimaerid fishes branching from the common cartilaginous antecedent around 420 mya [2].

Photosensitivity (light detection) is critical to the survival of many species, with cone- and rod-mediated vision being the best understood. Rods and cones represent the archetypal photoreceptors and are located within the (duplex) neural retina, where they sense both the intensity and wavelength of environmental light. Previous studies have shown that photopic (daylight) vision arose early in the evolution of the vertebrates and occurred prior to the divergence of the agnathans and gnathostomes [3], [4], [5]. The presence of five cone subtypes, each with distinctive spectral sensitivities, in the retina of the anadromous southern hemisphere (pouched) lamprey, *Geotria australis*, provides the potential for pentachromacy in this ancient group of jawless fishes [3], [4], [5], [6]. The adult pouched lamprey dwells in the brightly lit upper water column for more than six months of the year [7]. By contrast, the northern hemisphere sea lamprey, *Petromyzon marinus*, lives in a deeper aquatic environment (http://www.fishbase.org), where it is exposed to lower light intensities and a restricted region of the light spectrum, and has lost three out of the five cone photopigments [8]. Thus, it appears that the visual system of these ancient vertebrates possessed the ability to remodel itself in response to changes in both the spectral composition and intensity of light at different depths. Similar to the sea lamprey, the holocephalan chimaera, the elephant shark, *Callorhinchus milii* (also known as the elephant fish or ghost shark), dwells in deep water (200–500 m) off the continental shelves of southern Australia and New Zealand [9] and has lost both classes of short-wavelength-sensitive cone photopigments, SWS1 and SWS2 [10]. However, unlike *P. marinus*, the elephant shark returns to shallow estuarine waters (6–30 m) during the warmer months to spawn, which may account for the retention of the middle-wavelength-sensitive (rod opsin-like 2; RH2) and long-wavelength-sensitive (LWS) cone classes of photopigment, with a duplication of the *LWS* opsin gene, providing a total of three cone photopigments expressed within the retina of its large eyes [10].

Parallel to the visual system, many other photosensitive pigments exist that are involved in non-image-forming light detection. Like the visual photopigments, non-visual pigments consist of an opsin protein linked to the chromophore, retinal, a derivative of vitamin A_1_. One such pigment, melanopsin, has been described in some detail [11], [12], [13], [14], [15], [16], [17], [18]. In mammals, the detection of light for photoentrainment is restricted to the eye, where a small subset of retinal ganglion cells (RGCs) express “long” and “short” splice variants [16], [19], [20] of the mammalian form of melanopsin (OPN4M) photopigment [13], [20], [21], [22], [23] and recent evidence has shown that this mediates a number of photophysiological responses, including the regulation of circadian rhythms, sleep, pupil constriction and the production of melatonin [11], [12], [13], [14], [15], [17], [18]. Furthermore, the photopigment would appear to be bistable as it is able to form stable interactions with both *cis-* and *trans-*isomers of the retinal chromophore and, as such, resembles the invertebrate rhodopsins [24], [25], [26], [27].

In non-mammalian vertebrates, two evolutionary lineages of the melanopsin gene family exist, namely a mammalian-like (*opn4m*) class and a xenopus-like (*opn4x*) isoform [16], [28]. Unlike the ocular restriction in mammals, the tissue expression of melanopsin in non-mammals is varied and detectable not only in the retina (e.g. RGCs, horizontal cells (HCs), iris and the retinal pigment epithelium (RPE)), but also in the skin, deep regions of the brain and the pineal gland [28], [29], [30], [31], [32], [33], [34], [35], [36], [37], [38]. In the model teleost, the zebrafish (*Danio rerio*), the complement of *opn4* genes has recently been extended to five, with all copies expressed in the major layers of the retina, including in a subset of cone photoreceptors [38]. Melanopsins can exist as either bistable (like invertebrate rhodopsins) [24], [25], [26], [27] or monostable (like vertebrate cone and rod photoreceptors [39], [40]) isoforms that are biochemically distinct [38].

Despite the broad range of *opn4* expression in non-mammals, the functional significance of melanopsin is largely unexplored in these species. Similarly, little is known regarding this ancient photopigment in early vertebrates. Therefore, the aim of this study was to investigate (1) the presence and evolutionary origins and (2) the functional characteristics of melanopsin in a phylogenetically important deep-sea cartilaginous species, the elephant shark (*C. milli*).

## Materials and Methods

### Ethics Statement

Elephant sharks were caught off the coast of Western Port Bay, Victoria (Australia) by licensed commercial fishermen. The samples used in this study were taken from animals that were already dead when the fishermen returned to the fishing jetty. Pieces of tissue were taken from the dead fish, frozen and transported to the laboratory for the extraction of DNA and RNA.

### Detection of Elephant Shark Scaffolds Containing Melanopsin Gene Sequences

The presence of melanopsin sequences in the elephant shark was investigated by TBLASTN searching the 1.4X coverage elephant shark genome assembly [41], consisting of 640,131 scaffolds (http://esharkgenome.imcb.a-star.edu.sg/) with both chicken mammalian-like (OPN4M; GenBank accession number ABX10832) and xenopus-like (OPN4X; GenBank accession number ABX10830) melanopsin protein sequences as bait. Scaffold sequences that showed high levels of identity (*e*-value cutoff at 1×10^−7^) to either of the two chicken melanopsin orthologues were searched against the non-redundant protein database at NCBI (http://www.ncbi.nlm.nih.gov/) using BLASTX to confirm that they indeed code for melanopsin.

### Tissue Collection and RNA Extraction

To facilitate RNA analysis, such as the determination of full-length sequences and their expression, various elephant shark tissues were collected, flash-frozen in liquid nitrogen and stored at −80°C prior to RNA extraction. Total RNA was isolated using TRIzol reagent (Invitrogen, USA) according to the manufacturer’s protocol and treated with DNase I (Roche, USA) to eliminate any residual genomic DNA.

### 5′- and 3′-rapid Amplification of cDNA Ends (RACE)

The full-length cDNA sequences for three melanopsin genes were obtained by 5′- and 3′-RACE (Rapid Amplification of cDNA ends) using primers complementary to the predicted exonic sequences derived from analysis of the elephant genome. A total of 1 µg of total RNA was used for preparing single strand 5′- and 3′-RACE-ready cDNA using the SMART RACE cDNA Amplification kit (Clontech, USA). The single strand DNA was resuspended in 100 µl of Tricine-EDTA buffer and 1 µl was used as template for PCR. The PCR cycles comprised a denaturation step at 95°C for 2 min, followed by 35 cycles of 95°C for 30 s, 60°C for 30 s and 72°C for 2 min, followed by a final elongation step at 72°C for 5 min. 5′- and 3′-ends of cDNA were amplified in a nested PCR reaction according to the manufacturer’s instructions. Sequences of primers used for both 5′- and 3′-RACE are available on request. The PCR products were sequenced either directly or after cloning into pGEM-T vector (Promega, USA) to verify if any spurious mutations were introduced by the DNA polymerase. Sequencing was performed using a BigDye Terminator v3.1 Cycle Sequencing Kit (Applied Biosystems, USA) on an ABI 3730xl DNA analyser.

### Phylogenetic Analysis

A codon-matched nucleotide sequence alignment of three elephant shark melanopsin coding regions, opn4m1, opn4m2 and opn4xlong, was generated by ClustalW [42] and manually refined to compare opn4 sequences derived from a variety of chordates (lancelet to human) and a number of zebrafish visual and non-cone, non-rod opsin sequences. The nucleotide sequences for human GPR21 (GenBank accession number NM005294) and GPR52 (GenBank accession number NM005684) were used as outgroups and phylogenetic trees were generated using three different methods as follows: (1) A Bayesian Probabilistic Inference (BPI) method, with a Metropolis Markov chain Monte Carlo (MCMC) algorithm, was performed using MrBayes version 3.1.2 software [43], [44] that incorporated a general time-reversal (GTR) model [45] with a gamma-distributed rate of variation across all sites with a proportion of invariable sites. 2,700,000 generations (with a chain sample frequency = 1000) were executed in simultaneous rounds that began from two independent and random seed trees. To allow for tree convergence and a low (less than 0.01) standard deviation of split frequencies, the first 675 generations (25%) were discarded as burnin and the resultant tree was visualised by Treeview version 1.6.6 [46]; (2) Maximum Composite Likelihood (MCL) [47]; and (3) Kimura 2-Parameter (K2P) substitution [48] matrix methods were applied with pairwise deletion and a homogenous pattern (with uniform rates) of nucleotide substitution among lineages to generate a Neighbour-Joining (NJ) phylogenetic tree [49], bootstrapped with 1000 replicates to assess the degree of support for internal branching (expressed as a percentage). For (2) and (3), the MEGA Version 4.0.2 computer package [50] was used.

### Reverse Transcription Polymerase Chain Reaction (RT-PCR)

Due to the extreme difficulty in collecting multiple biological replicates of the elephant shark, quantitative PCR, or other similar methods of transcript quantification, could not be performed. However, an approximation of the relative levels of gene expression for the three elephant shark melanopsin sequences between tissues was determined by RT-PCR, using 1 µl of 5′-RACE-ready cDNA and the following primer combinations: Cm-opn4m1-RT-F (5′-GAGCCTGCGAACTCCAGCGAACAT-3′) and Cm-opn4m1-RT-R (5′-GTGAACGTCATGTAGTCCCAAGTG-3′); Cm-opn4m2-RT-F (5′-GACGGTAGATGTGCCAGACCAC-3′) and Cm-opn4m2-RT-R (5′-CTCCAGCCGAAGAAAGGAGGG-3′); and Cm-opn4x1-RT-F (5′-CAGTCTCCCGTCTTCTTCGTCAG-3′) and Cm-opn4x1-RT-R (5′-GCCAAACCAGAATGATGACCTTCAG-3′). The PCR cycles comprised a denaturation step at 95°C for 2 min, followed by 35 cycles of 95°C for 30 s, 60°C for 30 s and 72°C for 30 s, and a final elongation step at 72°C for 5 min. Actin was amplified using the primers Cm-actin-F (5′-GGTATTGTCACCAACTGGGAC-3′) and Cm-actin-R (5′-AGATGGGCACAGTGTGGGTG-3′) as a control for the quality of cDNA. Note that primer pairs were designed to span at least one intron to facilitate the distinction between cDNA and genomic DNA amplification.

### Construction of Elephant Shark Melanopsin Plasmids, Transfection and Cell Culture

Full-length sequences for elephant shark opn4m1, opn4m2 and opn4x were amplified as single fragments by PCR as previously described [8], [38], using either 5′-RACE-ready cDNA or previously cloned cDNAs as templates and the following primer combinations: Cm-opn4m1-PE-F (5′-GCGCGAATTCCACCATGAACGTTCATCCCATCAG-3′) and Cm-opn4m1-PE-R (5′-CGGCGTCGACGCCTTTGTAGTTTCCAAAATGGTG-3′); Cm-opn4m2-PE-F (5′-GCGCGAATTCCACCATGTTCCCGACGGTAGATGTG-3′) and Cm-opn4m2-PE-R (5′-CGGCGTCGACGCGCCAGGTCCTGGTCTACCTCTG-3′); and Cm-opn4x-PE-F (5′-GCGCGAATTCCACCATGGATTCCCACCACCGGACC-3′) and Cm-opn4x-PE-R (5′-CGGCGTCGACGCGACTATATGAGTTCCAAGTCC-3′). Once generated, each insert was digested with *Eco*RI and *Sal*I restriction enzymes and subcloned into the pMT4 expression vector [51], resulting in a fusion construct between each melanopsin cDNA and a short sequence encoding a carboxyl-terminal bovine rod opsin 1D4 epitope (ETSQVAPA) that would be recognised by the anti-Rho1D4 monoclonal antibody [52].

Neuro-2a cells (ECACC) were maintained in Dulbecco’s Modified Eagle’s Medium (DMEM) (Sigma, UK), supplemented with 10% foetal bovine serum (FBS) (Life Technologies, UK), 2 mM L-glutamine (Sigma, UK) and 1% (v/v) penicillin/streptomycin (Sigma, UK), in a humidified chamber at 37°C with 5% CO_2_. Cells were fed fresh media every 48–60 h and passaged prior to reaching confluence. Cells were trypsinised, diluted and cultured at 1×10^5^ cells per ml in 90 mm dishes for 24 h before transfection. Transient transfections were performed with plasmids containing full-length elephant shark melanopsin sequences fused in frame with a short sequence encoding the 1D4 epitope. GeneJuice transfection reagent (Novagen, UK) was used for all transfections and followed the manufacturer’s recommended protocol. In all cases, each melanopsin plasmid was co-transfected with a control pSIREN-DNR-DsRed-Express vector (Clontech, UK) for cell identification prior to electrophysiology. The day after transfection, cell differentiation was induced using 20 µM retinoic acid (Tocris, UK), and all cells were kept in complete darkness until electrophysiological analysis was performed 48–60 h post differentiation.

### Whole-cell Electrophysiology

Electrophysiological analyses were performed as previously described [25], [38]. Transfected cells were identified by mCherry fluorescence using a Zeiss Axioskop FS2 microscope. 20 µM 9-*cis* retinal (Sigma, UK) or all-*trans* retinal (Sigma, UK) was added to the perfusion solution (140 mM NaCl, 4 mM KCl, 1 mM MgCl_2_, 2 mM CaCl_2_, 5 mM glucose, 10 mM HEPES; pH 7.3–7.4, 24°C), and cells were kept in the dark for at least 1 h prior to recording. Ideally, 11-*cis* retinal (the predominantly utilised native chromophore) would be used instead of 9-*cis* retinal (commercially available analogue); however, due to the large amount of chromophore required, the latter was used for all electrophysiological experiments. Although the peak spectral sensitivity of pigments bound to 9-*cis* retinal is short-wavelength-shifted compared to those that form stable interactions with native 11-*cis* retinal, the use of either retinal isomer is interchangeable with regards to the activation of photopigments. Whole-cell patch-clamp recordings were made with pipettes containing 140 mM KCl, 10 mM NaCl, 1 mM MgCl_2_, 10 mM HEPES and 10 mM EGTA. Osmolarity was adjusted to 285±5 mosmol/L and pH to 7.3 to 7.4 with KOH. Open pipette resistance was 3.5–5 MΩ and access resistance during recordings was less than 20 MΩ. Currents were recorded (Axopatch 200B, Axon Instruments, USA) in neurons voltage clamped at holding potentials of −50 mV. The records were filtered at 1 kHz and sampled at 20 kHz. Light stimuli (420 nm) were generated using a Cairn Optoscan Xenon arc source comprising a slit monochromator. All stimuli were 10 s in duration with a 20 nm half-bandwidth. Irradiance was measured using an optical power meter (Macam Photometrics, UK) and converted to photon flux. The magnitude of the responses was defined by the peak-sustained current measured using Clampfit (Axon Instruments, USA).

## Results

### Identification, Phylogenetic Analyses and Expression of Elephant Shark Melanopsin Genes

**Figure 1:**
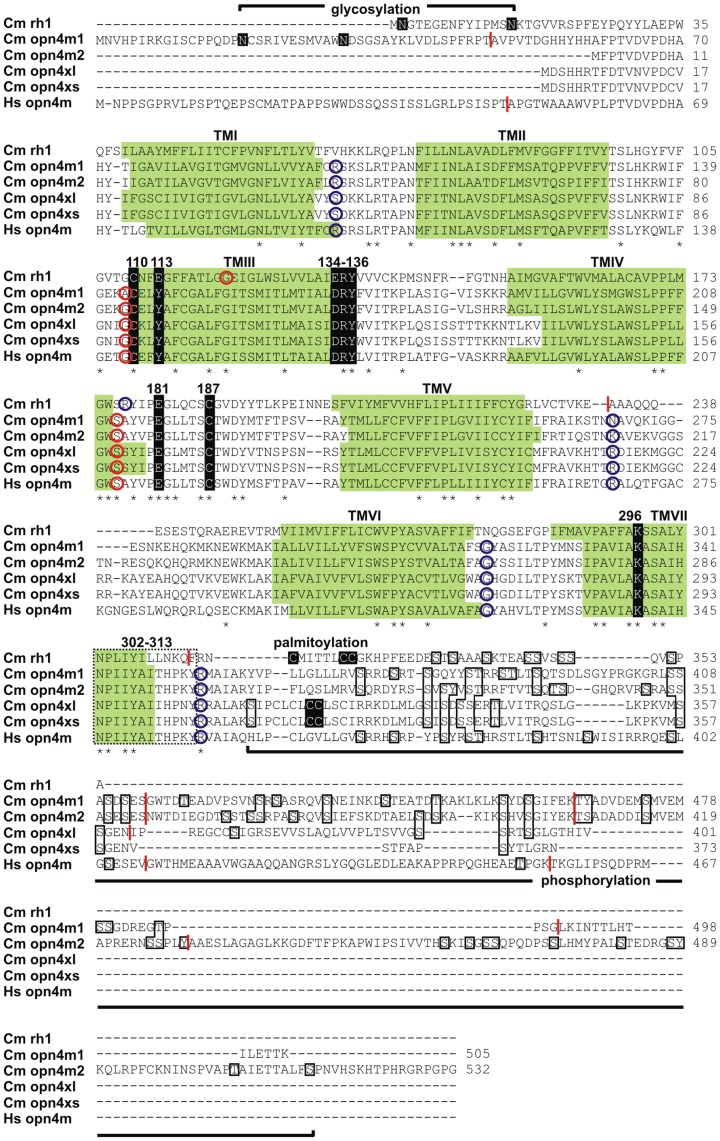
Elephant shark melanopsin sequences. An alignment of the four melanopsin polypeptides (opn4m1, opn4m2, opn4xlong and opn4xshort) expressed in the elephant shark (*Callorhinchus milii*, Cm) compared to the rod opsin (rh1) protein sequence from the same species (GenBank accession number: ABU84865) and the human (*Homo sapiens*, Hs) melanopsin (opn4m) polypeptide sequence (GenBank accession number: AAI43689). Identical amino acids (*) and gaps (–) present within the alignment are shown. Intron positions are shown as vertical lines or circles, where a red vertical line represents phase 0 introns (i.e. between two codons); a red circle marks the codons that are split in phase 1 (i.e. between the first and second bases of the codon); and a blue circle denotes codons that are split in phase 2 (i.e. between the second and third bases of the codon). Seven putative transmembrane (TM) domains for each elephant shark melanopsin protein were predicted using TMHMM Server Version 2.0 (http://www.cbs.dtu.dk/services/TMHMM/) and indicated by green shading. The TM domains for the elephant shark rod opsin were taken from [10]. Additional residues identified as being critical for correct opsin protein conformation and function are shown (highlighted in black or boxed) and discussed elsewhere [6], [16]. These include: (i) two putative glycosylation sites (Asn, N) that adhere to the consensus motif (Asn-X-Ser/Thr, N-X-S/T; X, any amino acid) for N-linked glycosylation present in the extracellular (EC) amino-termini of opn4m1 and opn4m2, but not in either isoform of opn4x (predicted online using NetNGlyc Server 1.0, http://www.cbs.dtu.dk/services/NetNGlyc/); (ii) two conserved cysteine (Cys, C) residues at positions 110 (TMIII) and 187 (EC2) that are involved in disulfide bond formation; (iii) a conserved tyrosine (Tyr, Y) at position 113 (TMIII), as found in many non-cone, non-rod opsins, instead of the glutamate (Glu, E) residue that serves as the negative counterion to the proton of the Schiff base for many visual opsins; (iv) a conserved glutamate (Glu, E) at position 134 (TMIII), located within a conserved Asp/Glu-Arg-Tyr (D/ERY) motif (134–136), that provides a negative charge to stabilise the inactive opsin molecule; (v) a conserved charged residue at site 181 that may serve as a counterion in many non-cone, non-rod opsins or affect spectral tuning; (vi) a conserved lysine (Lys, K) at position 296 (TMVII) that is covalently linked to a retinal chromophore via a Schiff base; (vii) a conserved Asn-Pro-X-X-Tyr-X_5,6_-Phe (NPxxY(x)_5,6_F) motif (302–313, dotted box) that assists in maintaining structural integrity upon photopigment activation; (viii) a small number of palmitoylation sites in both “long” and “short” isoforms of opn4x (and rh1), but absent in opn4m1 and opn4m2, that may be important in the tethering of the carboxyl-termini to the membrane (predicted online using CSS-Palm Server 3.0, http://csspalm.biocuckoo.org/online3.php [69]); and (ix) numerous potential serine (Ser, S), threonine (Thr, T) and tyrosine (Tyr, Y) phosphorylation sites in the carboxyl-terminal tails of all four protein isoforms that may be important in the regulation of phototransduction via the action of opsin-specific kinases (predicted online using NetPhos Server 2.0, http://www.cbs.dtu.dk/services/NetPhos/ [70]). All amino acids are conventionally numbered based on the bovine rod opsin protein (GenBank: accession number: NP001014890).

Mining of the elephant shark 1.4X genome assembly for sequences with high identity to vertebrate melanopsin (opn4) identified a number of scaffolds that were *opn4-like*. Based on these partial sequences, oligonucleotides specific for predicted exons were designed and used to generate initial partial cDNA sequences. Further primers were designed and subsequent 5′- and 3′-RACE experiments yielded full-length transcripts of three isoforms that are expressed in a number of tissues, including the eye. As the elephant shark assembly was generated with 1.4X coverage sequences, it is conceivable that other melanopsin genes may be present in the genome. However, since the large number of melanopsin-specific primer sets used for RT-PCR and 5′- and 3′-RACE experiments across a broad range of tissues yielded only the genes presented in this study, it would seem unlikely that other melanopsin genes remain to be identified. Melanopsins are divided into two gene classes based on their evolutionary origins and sequence divergence [28]. Subsequent BLASTX analyses suggested that the transcripts with coding regions of 1,515 bp and 1,596 bp were most similar to the “long” isoforms of the mammalian-like (*opn4m*) melanopsin gene class, while the third transcript (with a coding region of 1,206 bp) shared identity with the “long” isoforms of the xenopus-like (*opn4x*) gene class (Figure 1). Further PCR amplification analysis identified an additional transcript expressed in the snout of the elephant shark that on closer inspection was determined to be a splice variant of the opn4x-like transcript, differing only in its length and sequence at the carboxyl-terminus (73 amino acids compared to 101 residues for the longer isoform). Like melanopsin splice variants of other vertebrates, the alternative and shorter elephant shark opn4x transcript was generated by retention of the final intron rather than its removal by splicing. This partly processed transcript is present at lower levels than the fully processed form, with translation continuing to the first stop codon encountered within the intron (Figure 1) to give a total length of 374 amino acids.

The full-length coding sequences for all four elephant shark melanopsins are shown in Figure 1, with the predicted seven transmembrane domains highlighted together with all the residues critical for structural integrity and function, including conserved Cys110 and Cys187 residues for disulphide bond formation; a D/ERY motif at positions 134–136 for the stabilisation of the inactive opsin molecule; and a conserved lysine at site 296 (Lys296) required for the formation of the Schiff base between the opsin protein and the vitamin A-derived retinal chromophore. Note that the Glu113 “counterion” typical of visual opsins is replaced with Tyr113, a replacement that is common in non-visual pigments. In these latter opsins, it has been suggested that the counterion may be displaced to Glu181 [53], which is also conserved in the elephant shark melanopsin photopigments (Figure 1). Collectively, these data are consistent with the conclusion that all four elephant shark melanopsins are functional photopigments belonging to the G protein-coupled receptor superfamily. Further predictive biochemical analyses suggest the presence of structural and/or functional disparities between the melanopsin orthologues. These include the presence of two glycosylation sites in opn4m1 and opn4m2 isoforms, but their absence in both splice variants of the opn4x isoform (and in human opn4m); a double cysteine motif in both “long” and “short” opn4x isoforms that may be sites of palmitoylation, but omitted from all opn4m orthologues; and differing numbers and locations of putative phosphorylation sites in all four melanopsin isoforms (26 for opn4m1, 39 for opn4m2, 12 for opn4xlong and 9 for opn4xshort).

**Figure 2:**
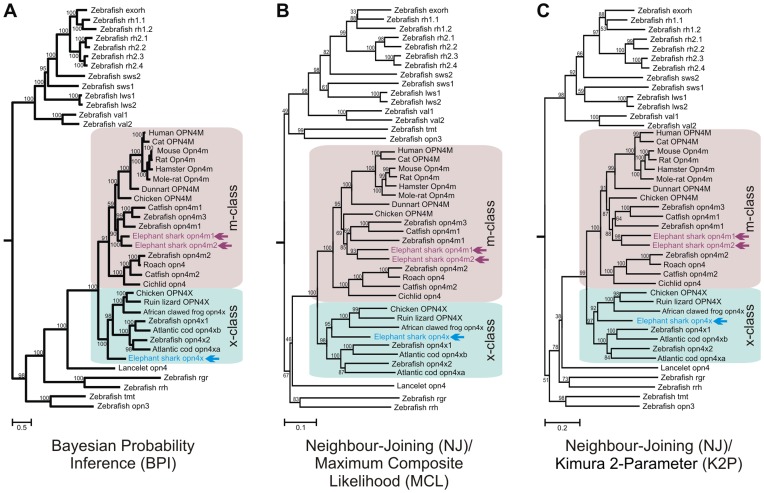
Phylogeny of melanopsin in *C. milii* and other vertebrates. Phylogenetic analyses based on a codon-matched nucleotide alignment of elephant shark opn4 cDNA sequences compared to the melanopsin sequences of representative chordates and the published visual and non-visual photosensory pigments of the zebrafish (*Danio rerio*), showing the relative positioning of elephant shark opn4m1, opn4m2 and opn4x photopigments (arrow) within the two main clades (m-class (purple) and x-class (blue)) of melanopsin. (**A**) A Bayesian Probabilistic Inference (BPI) method, performed with a Metropolis Markov chain Monte Carlo (MCMC) algorithm [43], [44] and incorporating a general time-reversal (GTR) model [45] with posterior probability values (represented as a percentage) indicated at the base of each node. (**B**) Maximum Composite Likelihood (MCL) [47] and (**C**) Kimura 2-Parameter (K2P) substitution [48] matrix methods, both generating bootstrapped, Neighbour-Joining (NJ) phylogenetic trees [49], with the degree of internal branching expressed as a percentage. The scale bar indicates the number of nucleotide substitutions per site. The human GPR21 (GenBank accession number: NM005294) and GPR52 (GenBank accession number: NM005684) nucleotide sequences were used as outgroups. The opsin sequences and their GenBank accession numbers used for generating the tree are as follows: (i) exorhodopsin (exorh): zebrafish (*Danio rerio*), NM131212; (ii) rod opsin (rh1): zebrafish (*Danio rerio*), NM131084 (rh1.1), HM367062 (rh1.2); (iii) rod opsin-like 2 (rh2): zebrafish (*Danio rerio*), NM131253 (Rh2.1), NM182891 (Rh2.2), NM182892 (Rh2.3), NM131254 (Rh2.4); (iv) short-wavelength-sensitive 2 (sws2): zebrafish (*Danio rerio*), NM131192; (v) short-wavelength-sensitive 1 (sws1): zebrafish (*Danio rerio*), NM131319; (vi) long-wavelength-sensitive/middle-wavelength-sensitive (lws/mws): zebrafish (*Danio rerio*), NM131175 (lws1), NM001002443 (lws2); (vii) vertebrate ancient (va) opsin: zebrafish (*Danio rerio*), AB035276 (va1), AY996588 (va2); (viii) panopsin (opn3): zebrafish (*Danio rerio*), NM001111164; (ix) teleost multiple tissue (tmt) opsin: zebrafish (*Danio rerio*), BC163681; (x) retinal pigment epithelium-specific rhodopsin homolog (rrh) (peropsin): zebrafish (*Danio rerio*), NM001004654; (xi) retinal G protein-coupled receptor (rgr): zebrafish (*Danio rerio*), NM001017877; (xii) mammalian-like melanopsin (opn4m): human (*Homo sapiens*), NM033282 (OPN4V1); cat (*Felis catus*), AY382594; mouse (*Mus musculus*), EU303118 (Opn4mlong); rat (*Rattus norvegicus*), NM138860; hamster (*Phodopus sungorus*), AY726733; mole-rat (*Spalax ehrenbergi*), AM748539; dunnart (*Sminthopsis crassicaudata*), DQ383281; chicken (*Gallus gallus*), EU124632 (OPN4Mlong); catfish (*Ictalurus punctatus*), FJ839437 (opn4m1), FJ839438 (opn4m2); roach (*Rutilus rutilus*), AY226847; cichlid (*Astatotilapia burtoni*), EU523855; zebrafish (*Danio rerio*), GQ925715 (opn4m1), GQ925716 (opn4m2), GQ925717 (opn4m3); elephant shark (*Callorhinchus milii*), JQ172797 (opn4m1), JQ172798 (opn4m2); (xiii) xenopus-like melanopsin (opn4x): chicken (*Gallus gallus*), EU124630 (OPN4Xlong); lizard (*Podarcis siculus*), DQ013043; African clawed frog (*Xenopus laevis*), AF014797; cod (*Gadus morhua*), AF385823 (opn4xa), AY126448 (opn4xb); zebrafish (*Danio rerio*), GQ925718 (opn4x1), GQ925719 (opn4x2); elephant shark (*Callorhinchus milii*), JQ172799 (opn4xlong); (xiv) chordate melanopsin (opn4): lancelet (*Branchiostoma belcheri*), AB205400; and (xv) outgroup (not shown): human (*Homo sapiens*), NM005294 (GPR21), NM005684 (GPR52). The gene nomenclature used follows the guidelines adopted by the Entrez Gene database (http://www.ncbi.nlm.nih.gov/sites/entrez?db=gene). In brief, the genes of all terrestrial species are in uppercase, except for the mouse and rat, where only the first letter is capitalized. The genes of all aquatic species, including amphibians, are in lowercase.

In order to determine the evolutionary relationships between the three elephant shark *opn4* genes, independent phylogenetic algorithms were employed, namely Bayesian Probability Inference (BPI) analysis and Neighbour-Joining (NJ) methodologies using both Maximum Composite Likelihood (MCL) and Kimura 2-Parameter (K2P) substitution matrices. Consistent with the initial bioinformatic analyses, all three phylogenetic methods showed that both elephant shark *opn4m-like* genes fall within the mammalian-like melanopsin class, whilst the opn4x isoform groups within the xenopus-like melanopsin class (Figure 2). These gene sequences, including the coding regions and 5′- and 3′-untranslated regions (UTRs), have been submitted to GenBank with the following accession designations: opn4m1 (JQ172797), opn4m2 (JQ172798), opn4xlong (JQ172799) and opn4xshort (JQ172800).

The opn4xlong and opn4xshort elephant shark transcripts share a nucleotide sequence identity of greater than 90%, which is consistent with their origin as splice variants derived from the same *opn4* gene. The monophyletic grouping of the elephant shark *opn4m1* and *opn4m2* genes, as sister groups to the three *opn4m-like* genes found in teleost fishes, suggests that this gene duplication occurred independently in the chimaera. It is possible that the monophyletic positioning of the elephant shark *opn4m1* and *opn4m2* genes arises from gene conversion, although both the nucleotide and amino acid sequences show only 56% identity, compared to 77% coding sequence identity for the two elephant shark long-wavelength-sensitive genes (*LWS1* and *LWS2*) that arose independently in the elephant shark lineage [10]. In many primates, a similar *LWS* gene duplication gave rise to *LWS* and middle-wavelength-sensitive (*MWS*) pigment genes (reviewed in [54]). In humans, gene conversion between these genes has resulted in an average coding sequence identity of 98% and an intron sequence identity of 100% (except for intron 1 where the sequence identity is 79%) [55]. Assuming that the elephant shark *opn4m1* and *opn4m2* genes shared a common ancestor with the *opn4m1*/*opn4m3* (an independent duplication that is restricted to many actinopterygians) and intronless *opn4m2* genes found in teleosts, it might be expected that the elephant shark genome may contain an intronless melanopsin gene. However, all of the elephant shark melanopsin genes identified in this study contain introns with positions that are conserved in *opn4* orthologues of other vertebrates (e.g. the human *opn4m* gene) and exhibit highly divergent sequences (Figure 1). Thus, based on the available data presented here, it would appear that the *opn4m1* and *opn4m2* genes of the elephant shark are limited to this lineage and represent, therefore, the first occurrence of a species-specific duplication of a melanopsin gene class for any organism so far studied; where duplicate *opn4* genes have been identified in other species [31], [37], [38], they are found throughout a phylogenetic Class or Order and not just in a single species. Nonetheless, this conclusion may be revised with the sequencing of melanopsin genes from other chondrichthyan species. In both cases, the *opn4m-like* and *opn4x-like* elephant shark orthologues are located at the base of their respective gene lineages, indicating that they derive from a gene duplication of a single ancestral melanopsin gene that is conserved presently in a chordate lancelet [24]. The elephant shark *op4x-like* genes are clearly related to the teleost *opn4x* class, but surprisingly, the *opn4m2* gene class identified in teleosts is located in a more ancestral position to the rest of the vertebrate melanopsin orthologues, suggesting that the gene duplication that gave rise to the intronless opn4m2 class occurred at the base of the jawed vertebrates (or perhaps earlier) and has been retained in teleost fishes, but lost subsequently in the cartilaginous fishes and the rest of the non-teleost bony vertebrates.

**Figure 3:**
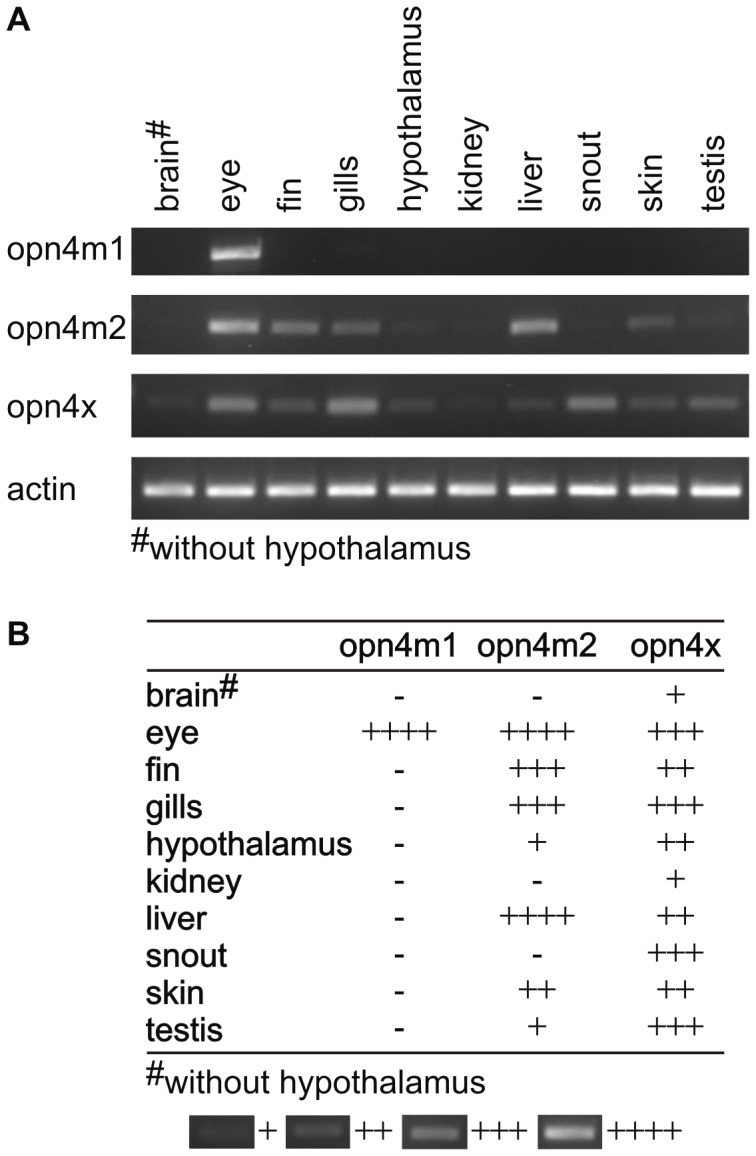
Expression of *opn4* genes in elephant shark tissues. (**A**) Relative expression of *opn4m1*, *opn4m2* and *opn4x* genes in a tissue panel of samples derived from the brain (without the hypothalamus), eye, fin, gills, hypothalamus, kidney, liver, snout, skin and testis of an adult elephant shark. Actin was included as a control for cDNA quality. (**B**) A summary table showing the relative patterns of melanopsin expression shown in the upper panel. In both panels, opn4m1 transcripts were shown to be limited to the eye, whereas both opn4m2 and opn4x (“long” and “short”) mRNAs exhibited broad expression across all the main tissue types investigated, including the adult eye.

To investigate the possible physiological requirement for multiple melanopsin genes in this deep-sea species, relative levels of expression were determined by reverse-transcription polymerase chain reaction (RT-PCR). As the “short” isoform of opn4x was restricted in its expression to the elephant shark snout, further analysis was not performed. The remaining three “long” isoforms were all relatively abundant in the eye of the elephant shark (Figure 3); opn4m1 was exclusively expressed in the eye, whereas both opn4m2 and opn4x transcripts were found in a wide-range of tissue types, including the brain (without the hypothalamus), eye, fin, gills, hypothalamus, kidney, liver, snout, skin and testis. Such diverse expression patterns are common for melanopsins in other species (e.g. teleosts) and suggest that melanopsin-based photosensitivity may be fairly wide-spread [28], [29], [30], [31], [32], [33], [34], [35], [36], [37], [38]), with putative roles in light-signalling and circadian photoentrainment of both central and peripheral clocks.

### Functional Characterisation of Elephant Shark Melanopsins

**Figure 4:**
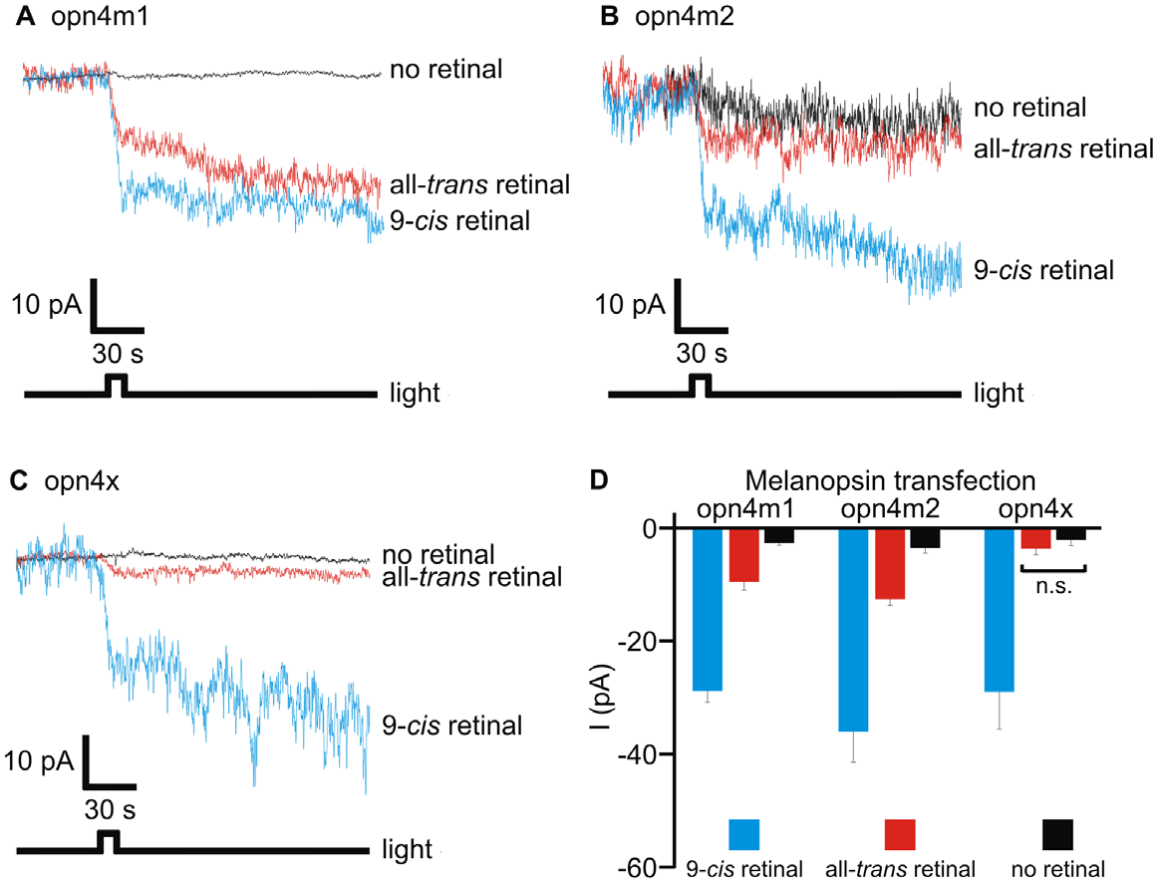
Functional analysis of elephant shark melanopsin photopigments. Light-evoked responses of neuro-2a cells expressing elephant shark melanopsins. Whole-cell recordings from cells expressing (**A**) opn4m1, (**B**) opn4m2 or (**C**) opn4xlong, preincubated with 9-*cis* (blue), all-*trans* (red) or no retinal (black) and exposed to a 10 s light pulse (8×10^14^ photons.cm^−2^.s^−1^) revealed that elephant shark melanopsins encode functional photopigments *in vitro* with *cis*-isomers of retinal chromophore, whereas only opn4m1 and opn4m2 exhibit light-evoked currents in the presence of all-*trans* retinal. (**D**) Quantification of melanopsin-dependent light-evoked currents with 9-*cis* retinal (*n* = 4–8 cells) (blue), all-*trans* retinal (*n* = 4–7 cells) (red) and no retinal (*n* = 4 cells) (black), respectively. Controls using untransfected cells in the presence of retinal were not light-responsive, with background currents comparable with no retinal experiments. Light-evoked currents (mean ± SEM) observed in cells expressing melanopsin preincubated with 9-*cis* retinal were significantly larger compared with cells with no retinal (*p*<0.05; Student’s *t*-test). Recordings with all-*trans* retinal showed that light-evoked responses of opn4m1- and opn4m2-transfected cells were larger (*p*<0.01; Student’s *t*-test) than those with no retinal. All possible permutations between two individual experimental values yielded significant differences (*p*<0.05; Student’s *t*-test), except for a comparison between the light-evoked currents determined for opn4xlong-transfected cells with or without all-*trans* retinal (0.05<*p*<0.5; Student’s *t*-test; denoted by n.s. (not significant)).

Both bistability (i.e. functional interactions with both *cis-* and *trans-*isomers of the retinal chromophore) and monostability (i.e. functional interactions with only the *cis-*isomer of the retinal chromophore) exist within the melanopsin classes in teleosts [38], whereas mammals and the lancelet orthologue possess melanopsin photopigments that are strictly bistable [24], [25], [26], [27], [56] and similar, therefore, to invertebrate rhabdomeric photopigments [57]. Although bistability is technically difficult to show directly, requiring multiple parallel experiments that investigate both the spectral characteristics and chromophore handling of a particular pigment, the formation of stable photopigments with different retinal chromophores is a strong indicator of a bistable nature. Such a case has been shown for the lancelet (*Branchiostoma belcheri*) pigment, Amphiop1, where this bistable pigment forms stable interactions with both 11-*cis* and all-*trans* retinal isomers, with a higher affinity for 11-*cis* retinal [57]. To determine whether the three elephant shark melanopsin genes encode functionally bistable or monostable photopigments, a heterologous expression system was used, as previously described for human OPN4 [25] and zebrafish opn4 [38]. Whole-cell patch-clamp recordings of neuro-2a cells that had been transiently transfected with each of the full-length melanopsin cDNA sequences revealed that elephant shark opn4 photopigments result in light-dependent inward currents in the presence, but not the absence, of 9-*cis* retinal chromophore (9-*cis* retinal: opn4m1, −29±5 pA; opn4m2, −36±15 pA; and opn4xlong, −29±11 pA) (Figure 4). When all-*trans* retinal was used, light-dependent inward currents were only observed in cells transfected with opn4m1 (−10±3 pA) and opn4m2 (−13±3 pA), with opn4xlong-transfected cells exhibiting similar background currents whether all-trans retinal was applied (−4±2 pA) or not (−2±2 pA). In all cases, the light-dependent responses showed very slow recovery, indicating that a native deactivation cascade may be absent in neuro-2a cells.

Consistent with similar experiments performed in zebrafish [38], the opn4m1- and opn4m2-derived currents measured with 9-*cis*-retinal were about 3-fold larger than those generated by *all*-trans retinal, suggesting that even though bistable photopigments may interact with multiple chromophores, the affinity of the interaction may differ between isoforms, and might be chromophore dependent and species-specific. Alternatively, the reduced light-activated current in the presence of all-*trans* may be due to endogenous activation/deactivation kinetics: for many bistable pigments, direct G-protein coupling/activation occurs in the presence of one form of chromophore, with minimal or no response when the isomerised retinal counterpart is present. However, in whole-cell electrophysiological experiments, where the net current that results from a steady-state level of activation/deactivation is the output measure, it is likely that all-*trans* retinal is photo-converted by bistable pigments into 11-*cis* retinal that activate the endogenous signaling cascade in a similar, albeit reduced, manner to experiments where *cis*-retinal isomers were used [25]. Whichever interpretation is correct, our functional studies indicate that the elephant shark opn4m1 and opn4m2 photopigments are likely to be bistable (similar to many chordate melanopsins [24], [25], [26], [27] and invertebrate rhabdomeric photopigments [57]) in their ability to form stable interactions with both 9-*cis* and all-*trans* chromophores, whereas opn4x elicits only an inward current with *cis*-isomers and is likely to be monostable (similar to a subset of teleost melanopsins [38] and classical vertebrate photopigments [39], [40]).

## Discussion

In vertebrates, the melanopsin genes fall into two independent sister lineages, a mammalian-like (*opn4m*) class and a non-mammalian-like or xenopus-like (*opn4x*) class [28]. All mammals so far studied, comprising the monotremes [16], the marsupials [20], [28] and many eutherians [16], possess an *OPN4M* gene that is expressed exclusively in the eye [16], [58], with splice-variants present in some species (e.g. mouse [20] and human [58]). The situation in non-mammalian species is far more complex, with many animals expressing two or more melanopsin splice-variants (e.g. chicken [19]) that differ in their carboxyl-termini. In many teleosts, melanopsin complexity is augmented by gene duplications that result in multiple copies of the *opn4* gene within each major class: examples include the five genes (*opn4m1*-*3* and *opn4x1*-*2*) in the zebrafish (*Danio rerio*) [38], two *opn4m* subtypes in the catfish (*Ictalurus punctatus*) [37] and *opn4xa* and *opn4xb* genes in the Atlantic cod (*Gadus morhua*) [31].

To investigate the evolutionary origins of these multiple melanopsin classes in early vertebrates, we have studied the elephant shark as a representative member of the Chondrichthyes, the phylogenetically important group that resides at the base of the extant gnathostomes. Two genes are present that belong to the *opn4m* (*opn4m1* and *opn4m2*) class and one to the *opn4x* class, with the latter having “long” and “short” isoforms. As these genes are sister groups to the duplications present in teleost fishes, it appears that only single forms of each class existed in early jawed vertebrates and these were retained in subsequent non-teleost species. Thus, the gene duplications that resulted in multiple melanopsin genes found in the Actinopterygii appear to be specific to that lineage and occurred after the divergence of the ray-finned fishes from the lobe-finned fishes at least 417 mya [59]. Nonetheless, like the teleosts, multiple class-specific melanopsin genes do exist in the genome of the elephant shark; however, the *opn4m1* and *opn4m2* genes appear to have arisen from an independent gene duplication event and are not, therefore, strict orthologues of the individual *opn4m1* and *opn4m3* genes found in teleost fishes. Whether this duplication is specific to the elephant shark or occurred in the common ancestor to all holocephalans, or even at the base of the chondrichthyan lineage, remains to be determined.

Many species that dwell in light-restricted environments, including the elephant shark, loose a subset of their visual photopigments [54]. Thus, it may be predicted that the complement of elephant shark melanopsin photopigments may be similarly affected by a reduction in the levels of irradiance. However, this is clearly not the case in this species, where a total of four *opn4* transcripts are expressed, which arise from the presence of orthologues from both lineages of melanopsin, gene duplication and alternative splicing.

Electrophysiological techniques have demonstrated that the elephant shark melanopsins exhibit both bistability and monostability biochemistries, as first shown in the zebrafish [38]. These appear to be class-specific: opn4x isoforms only function in the presence of *cis-*retinal, whereas opn4m pigments are able to interact with both *cis-* and *trans-*isomers of retinal. Despite a partial overlap in the chromophore handling between the elephant shark melanopsins, putative functional diversity is supported by comparisons between the amino acid sequences of each elephant shark melanopsin, where variations in post-translational modifications, for example in glycosylation, palmitoylation and phosphorylation, suggest that they may be structurally and functionally distinct. Moreover, each elephant shark *opn4* gene shows a different pattern of expression: each melanopsin gene is expressed in the eye, but opn4m2 and opn4x transcripts are, in addition, detectable in a broad range of other tissues that include skin, fin, gills, snout and liver. The significance of this differential pattern of expression is unclear, but may be related to the movement of the elephant shark from the deep ocean to shallow water in order to spawn, where it is exposed to a bright-light environment. Different melanopsins may be involved, therefore, in the spatial and temporal regulation of photoentrainment at these particular stages of the lifecycle. Further study is, however, required to determine the nature of any temporal changes in expression for each of the elephant shark melanopsins.

It is well-established that opsin-based visual photopigments (e.g. rod opsin, rh1) are shifted in many species to shorter wavelengths with increasing depth [60], [61] in response to changes in both the intensity and spectral composition of the available light [62], [63], [64]. Unlike the relatively rapid phototransduction kinetics exhibited by “classical” visual photoreceptors [65], melanopsin-based photoreceptive pathways in “higher” vertebrates (i.e. from teleosts to primates) appear to integrate photons over longer periods of time [66] and are mediated by pigments that are generally sensitive to shorter wavelengths that peak between 475 nm and 500 nm [19], [24], [26], [38]. It is possible, therefore, that a similar shift in the spectral absorbance of all or a subset of the elephant shark melanopsins might have occurred, with different isoforms becoming the key pigments in the shallow estuarine waters during the spawning season, whereas other variants may be more important at the deeper oceanic stages of the life cycle. The wavelengths of bioluminescent light emitted by marine species also change with depth, with spectral peaks around 490 nm, 470 nm, or 460 nm in shallow, midwater or deep water, respectively [67], [68]: an additional possibility is that different elephant shark melanopsins are specifically tuned to these wavelengths in order to monitor changes in marine bioluminescence as a cue for the photoentrainment of endogenous circadian or behavioural rhythms.
